# Comparison of Clinical Efficacy of Alectinib *Versus* Crizotinib in *ALK*-Positive Non-Small Cell Lung Cancer: A Meta-Analysis

**DOI:** 10.3389/fonc.2021.646526

**Published:** 2021-06-02

**Authors:** Hao Tang, Longyu Jin, Zhang Zhang, Zhibin Jiang, Zeeshan Malik

**Affiliations:** ^1^ Department of Cardio-Thoracic Surgery, The Third Xiangya Hospital, Central South University, Changsha, China; ^2^ Department of Cardio-Thoracic Surgery, Changsha Central Hospital, Changsha, China

**Keywords:** alectinib, crizotinib, *ALK* inhibitors, non-small cell lung cancer, efficacy and safety

## Abstract

**Objective:**

To systematically evaluate the efficacy and safety of alectinib versus crizotinib in the treatment of anaplastic lymphoma kinase (*ALK*) positive non-small-cell lung cancer.

**Methods:**

Studies about the efficacy of alectinib versus crizotinib in the treatment of *ALK*-positive non-small cell lung cancer were searched in PubMed, Scopus, Embase and the Cocharane Library from inception to February 15, 2020. Two reviewers independently screened these studies, extracted the data, assessed the risk of bias in the included studies by using the Cochrane risk assessment tool, and then used review manager 5.3 software for meta-analysis.

**Results:**

Three studies comprising a total of 697 patients with *ALK*-positive non-small cell lung cancer were included, 380 in the alectinib group and 317 in the crizotinib group. The dose of alectinib (300 mg) in J-ALEX were lower than the approved dose (600 mg), however the crizotinib group in all three studies received the recommended dose (250 mg). Performance bias was high in all three studies whereas, and the attrition bias was high in two studies (Toyoaki Hida 2017 and Solange peters 2017). The results of meta-analysis showed that: the overall response rate [OR = 2.07, 95% CI (1.41, 3.06), P = 0.0002], the progression free survival [HR = 0.34, 95% CI (0.21, 0.55), P <0.0001], the partial response [OR = 1.71, 95% CI (1.19, 2.46), P = 0.003], P = 0.001], in alectinib group were higher than that of crizotinib group. Though the total number of events in complete response and the disease control rate were more in alectinib group than that of crizotinib group, the meta-analysis results shows no significant differences between two drugs in the disease control rate [OR = 2.24, 95% CI (0.56, 8.88), P = 0.25], the complete response [OR = 1.82, 95% CI (0.75, 4.45), P = 0.19]. In addition, the number of events in the stable disease [OR = 0.45, 95% CI (0.28, O.74), P = 0.001], and the adverse events [OR = 0.50, 95% CI (0.23, 0.81), P = <0.0001] in alectinib group were lower than that of crizotinib group.

**Conclusion:**

Alectinib in terms of overall response rate, progression-free survival and partial response is superior to crizotinib in the treatment of *ALK*-positive non-small cell lung cancer and is well tolerated. Compared with crizotinib, alectinib is more effective than crizotinib and has a lower incidence of total adverse reactions. Meta-analysis results confirm the strong base for alectinib as a first-line treatment for *ALK*-positive NSCLC.

## Introduction

Lung cancer is the second most commonly occurring cancer worldwide accounting for 11.4% of the total new cancer cases. It was estimated that the number of new lung cancer cases in the world exceed 2.2 million in 2020, second only to breast cancer. In many countries, lung cancer is currently the leading cause of cancer deaths, and accounting for approximately 20% of all cancer death rate. Lung cancer deaths in China is comparatively high compared to most countries (1). It is foreseen that lung cancer deaths in China may increase by roughly 40% between 2015 and 2030 (2). By 2017, the incidence of lung cancer in China had risen to 800,000 cases, while the mortality had reached 700,000. This shows that China’s primary bronchial lung cancer morbidity and mortality have an alarming growth rate, non-small cell lung cancer (NSCLC) is the most common form of lung cancer, accounting for approximately 85% of all lung cancer cases (3, 4). For early stage (I, II) NSCLC, surgery is the best treatment. But NSCLC is usually advanced at the time of diagnosis, and systemic treatment is its mainstay of treatment (5, 6). As it is well known that, many patients with advanced NSCLC benefit from chemotherapy to a certain level. Platinum-based chemotherapy is the standard treatment for patients with advanced NSCLC (7). However, chemotherapy and radiotherapy often has more side effects. Moreover, the 5-year survival rate of NSCLC is still below 27% (8). Hence, due to aforementioned reasons, we need more and better treatment strategies for advanced NSCLC.

In recent years, a meta-analysis result showed that crizotinib is more effective than chemotherapy in treating anaplastic lymphoma kinase (*ALK*) positive advanced NSCLC (9). Crizotinib is ALK’s first small molecule inhibitor and was approved in the US in 2011 for the treatment of patients with *ALK*-positive advanced NSCLC (10). However crizotinib resistance occurs, often within 12 months of the start of treatment ultimately resulting in disease progression (11). Due to crizotinib resistance the second generation alectinib was developed and get approval by US drug and food administration (FDA) in 2015 (12). The three recently conducted studies (11–13) exhibited that the alectinib is more effective than crizotinib.

In 2007, researchers found a fusion gene of echinoderm microtubule-associated protein-like protein 4 (*EML4*) and *ALK* in NSCLC tissue specimens (14, 15). The activated *ALK* fusion protein leads to abnormal ALK signaling through several molecular signaling pathways, including PI3K/AKT/mTOR, JAK/STAT, and RAS/MEK/ERK, and finally leads to cancer. According to statistics, about 3–7% of patients with NSCLC have *ALK* gene rearrangement, and it is more common in young patients with adenocarcinoma and patients who have never or have a slight history of smoking (16).

In more than 13 years of discovery of NSCLC containing *ALK* gene mutations, scientists are devoted to the development of ALK inhibitors. Currently, five ALK inhibitors have been approved by the FDA for *ALK*-positive advanced NSCLC including crizotinib, alectinib, ceritinib, brigatinib and lorlatinib.

Alectinib once was used as second line treatment of crizotinib-resistant patients, but now it is recommended as the first-line therapy in *ALK*-positive NSCLC. Crizotinib affirmed by FDA in 2011 is still the first-line treatment standard in numerous locales of the world due to the adequacy illustrated within the randomized stage III clinical trial when compared with platinum-based chemotherapy, in terms of both by overall response rate (ORR) and progression-free survival (PFS) (17, 18). Despite of better results of crizotinib compared with platinum based chemotherapy, resistance to crizotinib finally occurs more often within 12 months of beginning of treatment (19, 20).

Next-generation, alectinib, a highly selective central nervous system (CNS)-active ALK inhibitor, was developed to confer resistance to crizotinib (21, 22). Alectinib was approved by FDA in 2015. To assess its clinical efficacy, alectinib versus crizotinib comparative ALEX trial was conducted, in which alectinib shows superiority over crizotinib in terms of ORR, PFS and toxicity profile (13, 23, 24). To further evaluate the efficacy of alectinib another comparative study J-ALEX was conducted, which continued to show superiority of alectinib over crizotinib, PFS (34.1 vs 10.2 months; HR 0.80), median OS not reached alectinib vs 43.7 months crizotinib, and toxicity profile (adverse events grade ≥3 (36.9% *vs* 60.6% crizotinib) (12, 25). Another, recently conducted comparative study of alectinib versus crizotinib (ALESIA), shows better results in favor of alectinib ORR (91% *vs* 48%), PFS (not reached *vs* 11.1 months), and grades 3–5 adverse events (29% *vs* 48%) (11).

Due to the significant efficacy shown in the phase I clinical trial, ceritinib was approved by FDA in 2014 for the treatment of patients with *ALK*-positive metastatic NSCLC that progressed or could not be tolerated after crizotinib treatment. Ceritinib showed superiority to standard of care platinum-pemetrexed chemotherapy in the phase III ASCEND-4 trial (ORR, 72.5% *vs* 26.7%; PFS, 16.6 *vs* 8.1 months). This agent also demonstrates essential intracranial and extra cranial activity. Unluckily, the toxicity profile of ceritinib can limit its clinical utility. Within the significant randomized trial, the predominance of measurements alterations or interruptions was 80% within the ceritinib arm compared with 45% within the chemotherapy arm, separately (26, 27). In ASCEND-5 trial ceritinib appears longer PFS (5.4 vs 1.6 months; HR 0·49; p <0·0001), 43% in ceritinib shows serious adverse events (AEs) while 32% in chemotherapy (28). However, in single-arm trial comparative study of alectinib versus ceritinib, median OS with alectinib was prolonged 24.3 *vs* 15.6 with ceritinib; HR: 0.65; 95% CI: 0.48–0.88 (29). It was also confirmed by the recently published cross-study indirect comparison, which demonstrated 22% lower hazard ratio compared to ceritinib; HR: 0.78 (30).

On April 28, 2017, Brigatinib got approval by the US FDA for use in patients with *ALK*-positive metastatic NSCLC who are intolerant to crizotinib or whose disease has progressed after treatment. In recently conducted phase 3 ALTA-1L trial comparing brigatinib versus crizotinib, 275 patients were randomized; brigatinib (n = 137), crizotinib (n = 138), 26% patients in brigatinib while 27% patients in crizotinib group earlier received chemotherapy for advanced disease, 29% (brigatinib)/30% (crizotinib) had baseline brain metastases, at the data cutoff median follow-up was brigatinib/crizotinib: 11.0/9.25 months; BIRC-assessed PFS (HR 0.49, 95% CI 0.33, 0.74, p = 0.0007), brigatinib PFS (not reached *vs* 9.8 months), investigator-assessed PFS (HR 0.45 (95% CI 0.30, 0.68), p = 0.0001), most common treatment related adverse events (AEs) with brigatinib were elevated creatine phosphokinase (CPK) (16.2%), elevated lipase (13.2%), hypertension (9.6%); crizotinib: increased ALT (9.5%), AST (5.8%), and lipase (5.1%). Any grade ILD/pneumonitis: brigatinib, 3.7%; crizotinib, 2.2%. Discontinuations due to AEs (brigatinib/crizotinib): 11.8%/8.8% (31). There is another ongoing study comparing efficacy of brigatinib versus alectinib with an expected duration of five years to obtain the final results of the trial (NCT03596866).

Third generation ALK inhibitor Lorlatinib approved by US FDA in 2018. The efficacy of lorlatinib was then confirmed in a global phase II trial in patients with *ALK*- or *ROS1*-positive advanced NSCLC (32). Based on *ALK* and *ROS1* status as well as on pretreatment, patients were enrolled into six different expansion cohorts, 100 mg dose was prescribed once a day, patients (n = 276) had been listed in one of the following groups, ALK treatment naive (n = 30; EXP1), 59 who were *ALK* positive and received previous crizotinib without (n = 27; EXP2) or with (n=32; EXP3A), previously received one non-crizotinib ALK inhibitor with or without chemotherapy (n = 28, EXP3B), 112 who were *ALK* positive with two (n = 66; EXP4) or three (n = 46; EXP5) previous ALK inhibitors with or without chemotherapy, 47 who were ROS1 positive with any previous treatment (EXP6). Among *ALK*-positive patients, the OR was 90% for treatment-naive patients (EXP1) and 47% for those with at least one previous ALK TKI (n = 198; EXP2-5), Intracranial responses were seen in 2/3 (67%) treatment-naïve patients and 51/81 (63%) patients pretreated with at least one ALK TKI, in patients with only crizotinib pretreatment (EXP2-3A) responses were 69.5% (41/51), 9/28 (32.1%) patients with one previous non-crizotinib ALK TKI (EXP3B), and 43/111 (38.7%) patients with two or more previous ALK TKIs (EXP4-5). Intracranial responses were seen in 20/23 (87%) patients in EXP2-3A; 5/9 (55.6%) patients in EXP3B; and 26/49 (53.1%) patients in EXP4-5. Treatment-related adverse events were hypercholesterolemia (81% of patients; 15% grades 3–4), hypertriglyceridemia (60%; 16% grades 3–4), edema (43%; 2% grades 3–4) and peripheral neuropathy (30%; 2% grades 3–4). Weight gain was common with 10–20% increase in 31% of patients. Serious treatment-related adverse events were seen in 7% of patients. Lorlatinib is currently also compared with crizotinib in previously untreated patients within a randomized trial (NCT03052608) (33). Due to its late approval, there are no clinical studies comparing alectinib and other ALK TKIs.

Alectinib is first-line ALK TKI due to its PFS advantage, brain metastasis cumulative incidence reduction and favorable toxicity profile, for *ALK*-positive stage III or IV NSCLC, the current NCCN guidelines preferred the alectinib as first-line drug therapy (34). At present, there is no meta-analysis of the efficacy comparison between Alectinib and Crizotinib, we combined three studies to evaluate the systemic efficacy and safety of Alectinib versus Crizotinib to provide the further reliable basis for Alectinib as the most recommended first-line medication for *ALK*-positive stage III or IV NSCLC.

## Materials and Methods

### Inclusion Criteria

#### Type of Study

Randomized controlled trial (RCT), Clinical Controlled Trial (CCT), retrospective analysis.

#### Research Objective

*ALK*-positive patients with advanced (stage IIIB or stage IV) NSCLC. ECOG or WHO score is 0–2 points.

#### Intervention

The experimental group was treated with Alectinib. The control group was treated with Crizotinib.

#### Outcome Indicators

Overall response rate (ORR), progression-free survival (PFS), disease control rate (DCR), complete response (CR), partial response (PR), stable disease (SD), adverse events (AEs).

### Exclusion Criteria

① One-arm study, ② Meeting report, ③ News, ④ Republished research, ⑤ studies not reporting outcome of our interest. ⑥ Studies where data is difficult to extract.

### Literature Retrieval Strategy

Computer search of four databases: PubMed, Scopus, Embase and the Cocharane Library. We search for relevant clinical studies on the efficacy of Alectinib compared with Crizotinib in treating *ALK*-positive advanced NSCLC. The last search was performed on February 15, 2020. The aforementioned online data basses were systematically searched with one or combination of the following terms: “Lung Neoplasms”, “Carcinoma, Non-Small-Cell Lung”, “*ALK*-positive”, “Alectinib”, and “Crizotinib.” Mesh terms and free terms were used for each search and there were no restrictions based on language. The search strategies and results were recorded and uploaded as **Supplementary Material**. In addition, the reference lists of included studies were also manually searched to hunt potentially eligible articles.

### Literature Screening and Data Extraction

The two researchers independently screened the literature, extracted the materials needed for this study, and exchanged the results with each other. If the opinions of the two were not consistent, they asked for the intervention of a third party. According to the third party’s opinion, the three parties discuss together to solve the problem, and try to supplement the lack of information through other methods, such as contacting the author directly to obtain it. During literature screening, duplicate documents were deleted first, and then the titles and abstracts were quickly assessed. After excluding documents that were not significantly related to the study, the full-text of the remaining documents were thoroughly assessed, and finally it was determined whether to include in the study.

The contents of the data extraction mainly include: ① the basic characteristics of the included research, including the article title, the time of publication, and the first author; ② the basic characteristics of the research object, such as the number of samples in each group, whether they smoke, and ethnicity; ③ specific intervention measures, such as which ALK inhibitor to use and its usage and dosage, etc. ④ Specific elements of bias risk assessment; ⑤ outcome indicators.

### Literature Quality Evaluation

Two researchers used the Cochrane risk bias assessment tool to independently generate random distributions of the literature, allocate concealment, blind to researchers and subjects, blind to outcome indicators, completeness of outcome data, selective reporting studies. The results and other sources of bias are evaluated for the risk of bias. If the opinion of the two parties was not consistent, a third party’s opinion was sought and discussed and resolved.

### Statistical Analysis

Meta-analysis was performed using RevMan 5.3 software. The two categorical variables use odd ratio as the effect indicator, and the time survival variable uses HR as the effect indicator. Each effect amount gives its point estimate and 95% CI. Heterogeneity among the included studies was judged by P and I^2^ values. If there is no significant statistical heterogeneity between the results of each study (I^2^ ≤50%, P ≥0.1), a meta-analysis is performed using a fixed-effects model; if statistical heterogeneity exists between the results of each study (I^2^ >50%, P <0.1), then further analyze the source of heterogeneity, after excluding the effects of obvious clinical heterogeneity, use a random effects model for meta-analysis, the test level of the meta-analysis was set to p = 0.05.

## Results

### Study Selection

**Figure 1:**
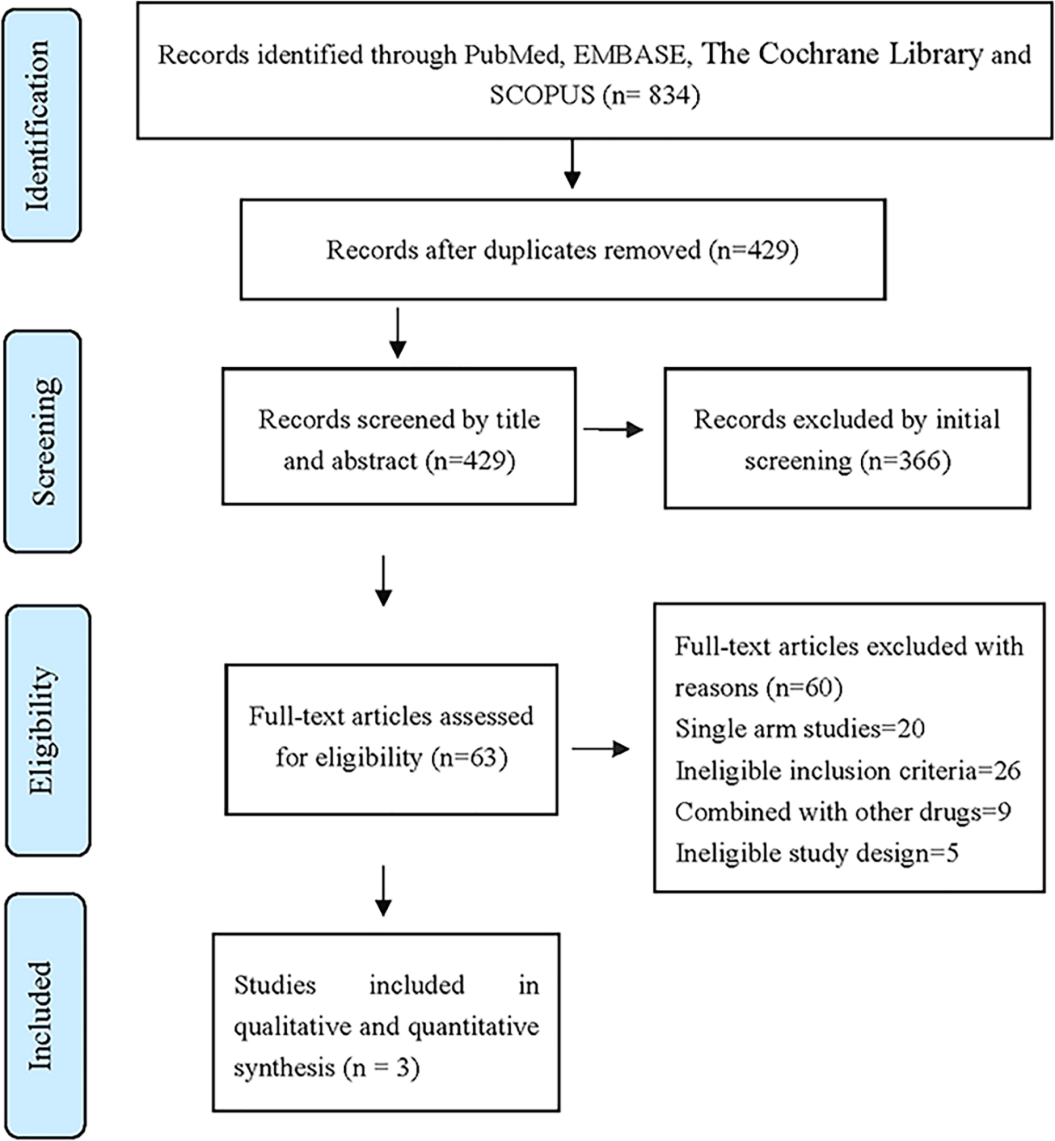
The database retrieved by 834 articles and the number of documents detected are as follows: PubMed (n = 158), Embase (n = 360), Scopus (n = 270), The Cochrane Library (n = 46).

After layer-by-layer screening according to the inclusion and exclusion criteria mentioned in **Table 1**, a total of 834 articles were retrieved from four databases: PubMed, Scopus, Embase and the Cocharane Library. Three articles (11–13) were finally included. The three articles were all in English. A total of 697 patients with *ALK*-positive advanced NSCLC were included, 380 in the Alectinib group and 317 in the Crizotinib group. The literature screening process and results are shown in **Figure 1**.

**Table 1:** Study Eligibility Criteria.

Level 1 Screening Questions (Title and Abstract)
● Covidence screening based on exclusion criteria
1) Exclude if any study does not look at ALK-positive NSCLC. 2) Exclude if the comparison of alectinib versus crizotinib includes other ALK inhibitors or other treatment options such as compare with chemotherapy, radiotherapy, immunotherapy, etc. in ALK-positive NSCLC patients. 3) Exclude if any study is not assessing the effectiveness of comparison of Alectinib versus Crizotinib in terms of Progression-free survival (PFS), Overall response rate (ORR), Complete response (CR), Disease control rate (DCR), Partial response (PR). Stable disease (SD), Adverse events (AEs). 4) Exclude if the study is not a primary study. 5) Exclude if the study is not in English. 6) Exclude if the study is not a comparative study.
Level 2 Screening Questions (Full Text)
● Covidence screening based on exclusion criteria.
1) Exclude the study if it consists of the following combined drug therapy: Alectinib versus Crizotinib combined with other ALK inhibitors. Alectinib versus Crizotinib combined with chemotherapy. Alectinib versus Crizotinib combined with Immunotherapy. Alectinib versus Crizotinib combined with radiotherapy. 2) Exclude if the study does not compare Alectinib versus Crizotinib in ALK-positive NSCLC.

### Basic Characteristics of Included Studies and Evaluation of Literature Quality

**Figure 2:**
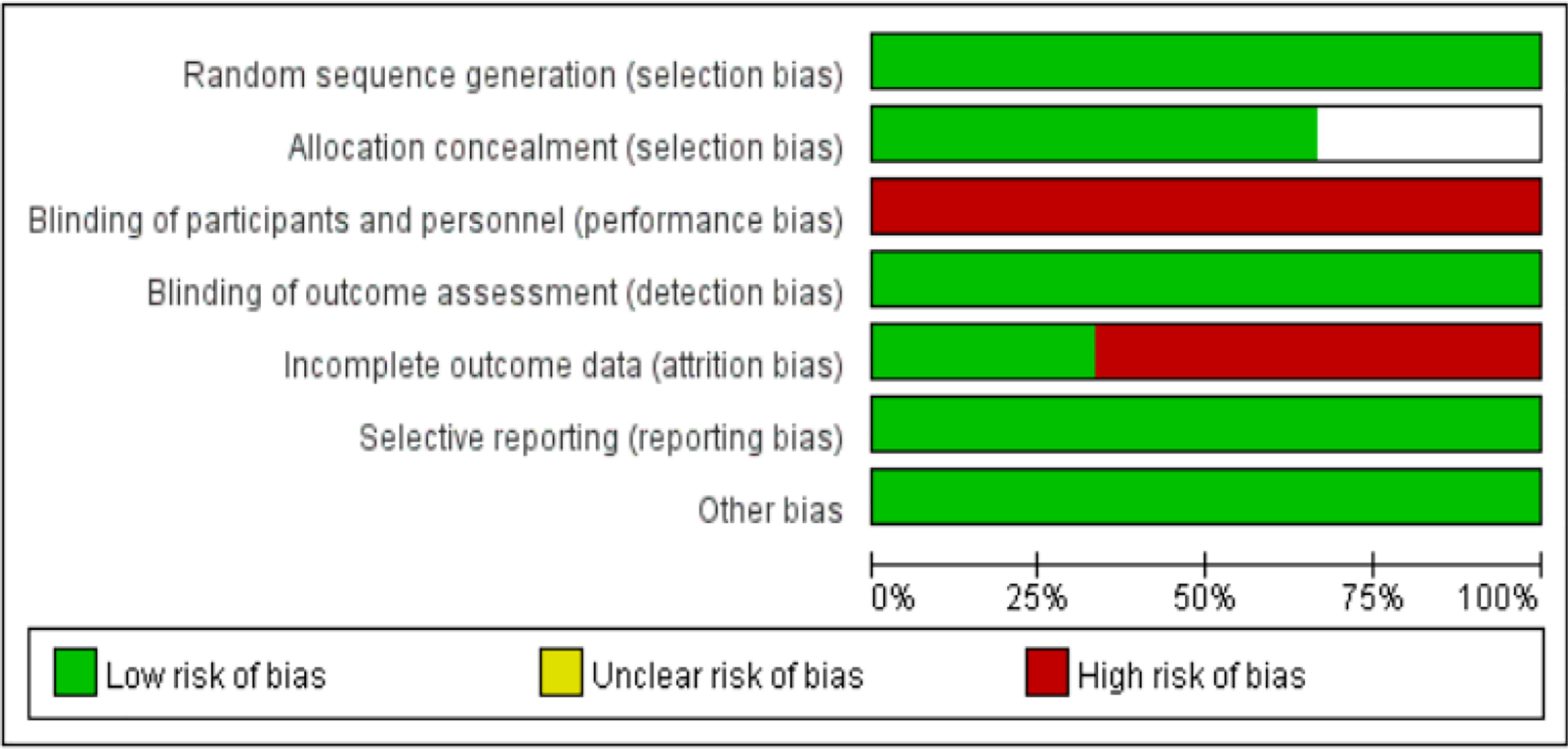
Risk of bias assessment tool for included studies.

**Figure 3:**
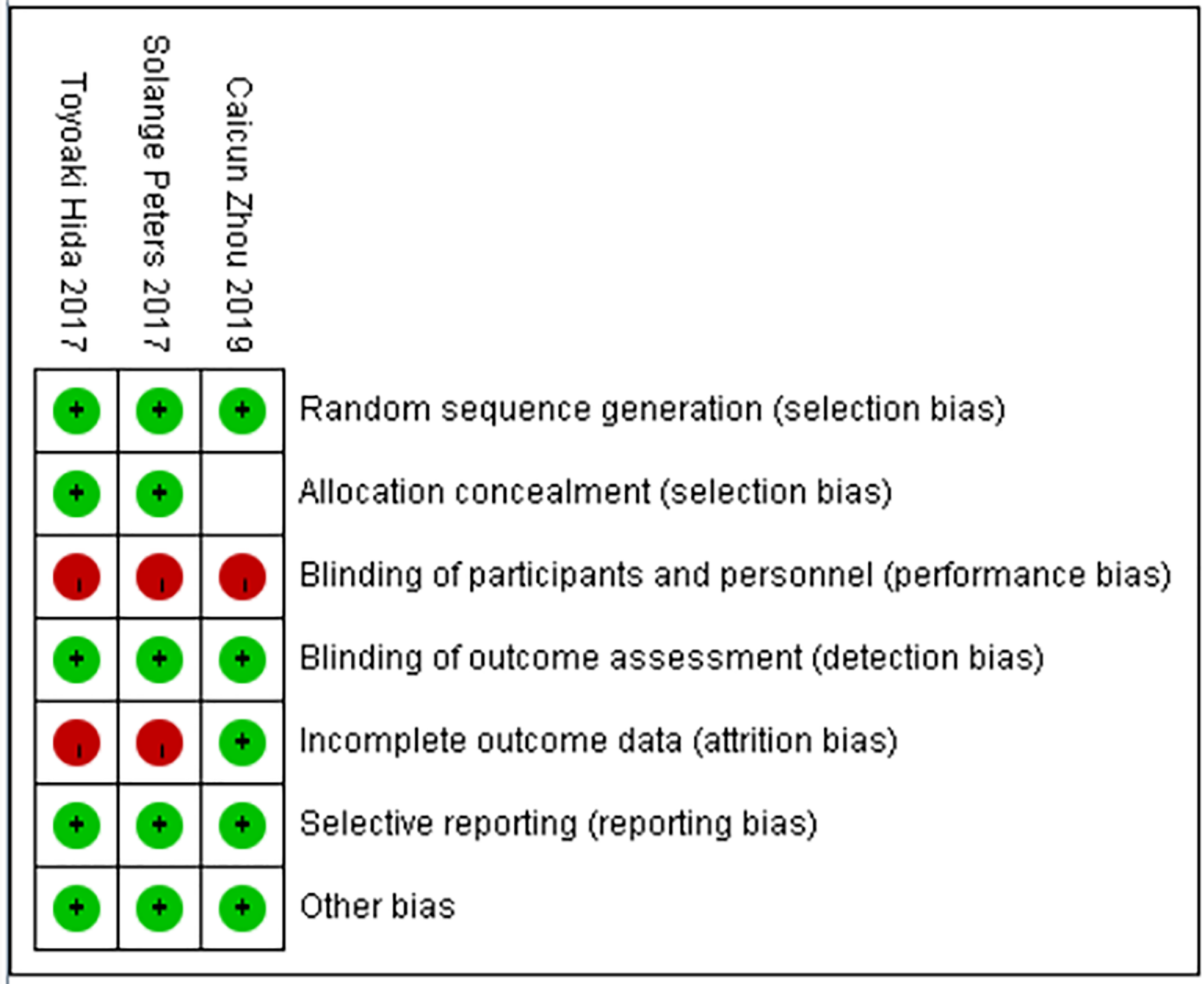
Summary of risk of bias in included studies.

The basic characteristics of the included studies are shown in **Tables 2** and **3**. The bias risk assessment tables included in the study are shown in **Tables 4** and **5**. The bias risk map for the included studies is shown in **Figure 2**. A summary of the risks of bias included in the study is shown in **Figure 3**.

**Table 2:** Basic characteristics of included studies.

Type of Studies (RCT)	Number of samples		Age (Years)		Sex	
	Experimental Group	Control Group	Experimental Group	Control Group	Male	Female
Toyoaki Hida 2017 (12)	103	104	60 (median)	59.5 (median)	82	125
Solange Peters 2017 (13)	152	151	58 (median)	54 (median)	132	171
Caicun Zhou 2019 (11)	125	62	51 (median)	49 (median)	98	89

**Table 3:** Basic characteristics of included studies.

Included Studies	Patients had received systemic treatment before enrollment?	Intervention measures Experimental group	Control group
Toyoaki Hida 2017 (12)	Uncertain	Alectinib 300 mg, Bid	Crizotinib 250 mg, Bid
Solange Peters 2017 (13)	NO	Alectinib 600 mg, Bid	Crizotinib 250 mg, Bid
Caicun Zhou 2019 (11)	NO	Alectinib 600 mg, Bid	Crizotinib 250 mg, Bid

**Table 4:** Risk of bias in included studies.

Included Studies	Random sequence Generation	Allocation Concealment	Blinding of participants and personal
Toyoaki Hida 2017 (12)	Low Risk	Low Risk	High Risk
Solange Peters 2017 (13)	Low Risk	Low Risk	High Risk
Caicun Zhou 2019 (11)	Low Risk	Low Risk	High Risk

**Table 5:** Risk of bias in included studies.

Included Study	Blinding of outcome Assessment	Incomplete outcome Data	Selective Reporting	Other Bias
Toyoaki Hida 2017 (12)	Low Risk	High Risk	Low Risk	Low Risk
Solange Peters 2017 (13)	Low Risk	High Risk	Low Risk	Low Risk
Caicun Zhou 2019 (11)	Low Risk	Low Risk	Low Risk	Low Risk

### Meta-Analysis Results

#### Overall Response Rate (ORR)

**Figure 4:**
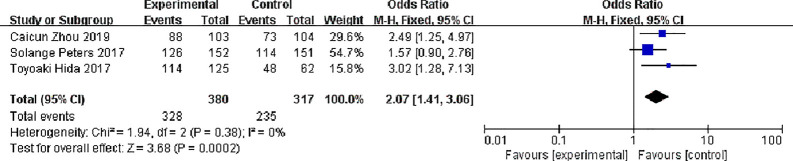
Meta-analysis of alectinib group and crizotinib group overall response rate.

Three studies reported total response rates, There were 380 patients in alectinib and 317 patients in the crizotinib, The study indicates very low statistical heterogeneity (P = 0.38, I^2^ = 0%), Due to low heterogeneity among the studies, the fixed effect model was used for Meta-analysis. Meta-analysis results shown in **Figure 4** suggest that Overall response rate of the alectinib was higher than that of crizotinib, and the difference was statistically significant. [0R = 2.07, 95%CI (1.41, 3.06), P = 0.0002].

#### Progression-Free Survival (PFS)

**Figure 5:**
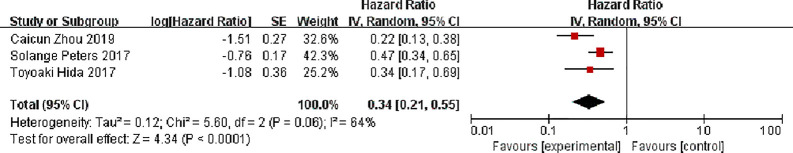
Meta-analysis of alectinib group and crizotinib group progression-free survival.

Three studies all reported progression-free survival, There were 380 patients in the alectinib and 317 patients in the crizotinib, with moderate statistical heterogeneity between the studies (P = 0.06, I^2^ = 64%), but there was an obvious clinical heterogeneity, and a random effect model was used for meta-analysis. Meta-analysis results shown in **Figure 5** suggest that the progression-free survival of the alectinib was higher than that of crizotinib, and the difference was statistically significant [HR = 0.34, 95%CI (0.21, 0.55), P <0.0001].

#### Disease Control Rate (DCR)

**Figure 6:**
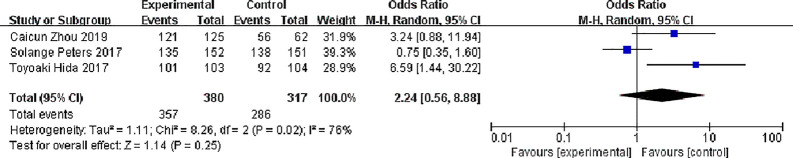
Meta-analysis of disease control rate between the alectinib group the crizotinib group.

Three studies reported disease control rates. There were 380 patients in the alectinib group and 317 patients in the crizotinib group. There was slight statistical heterogeneity between the studies (P = 0.02, I^2^ = 76%), but there was no obvious clinical heterogeneity, and a random effect model was used for meta- meta-analysis. Meta-analysis results shown in **Figure 6** suggest that the difference was not statistically significant, indicating no difference in DCR between the two drugs [OR = 2.24, 95% CI (0.56, 8.88), P = 0.25].

#### Complete Response (CR)

**Figure 7:**
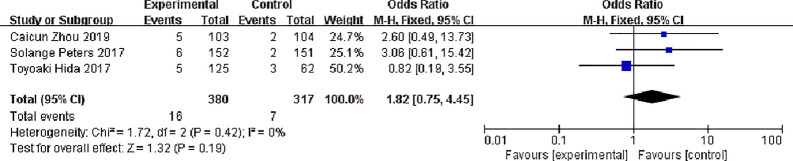
Meta-analysis of complete response rate between alectinib group and crizotinib group.

Three studies reported complete response rates, 380 patients in the alectinib group and 317 in the crizotinib group, with no significant statistical heterogeneity between the studies (P = 0.42, I^2^ = 0%), Meta-analysis was performed using a fixed effects model. Meta-analysis results shown in **Figure 7** suggest that the difference was not statistically significant, indicating no difference in CR between the two drugs [OR = 1.82, 95% CI (0.75, 4.45), P = 0.19].

#### Partial Response (PR)

**Figure 8:**
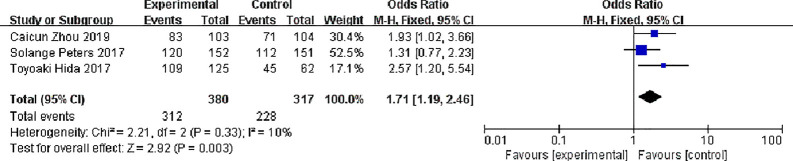
Meta-analysis of partial response rate between alectinib group and crizotinib group.

Three studies reported partial response rates, 380 patients in the alectinib group and 317 patients in the crizotinib group, with slight statistical heterogeneity between the studies (P = 0.33, I^2^ = 10%), but no obvious clinical heterogeneity, a fixed effect model was used for meta-analysis. Meta-analysis results shown in **Figure 8** suggest that the partial response rate of alectinib group was higher than that of crizotinib group, and the difference was statistically significant [OR = 1.71, 95% CI (1.19, 2.46), P = 0.003].

#### Stable Disease (SD)

**Figure 9:**
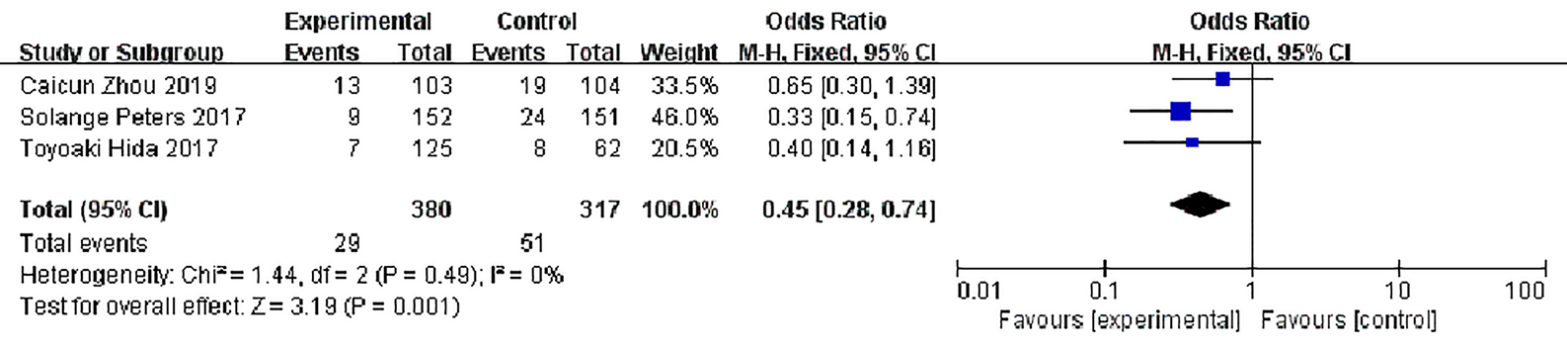
Meta-analysis of disease stability rate between the alectinib group and crizotinib group.

Three studies reported disease stability rates, 380 patients in the alectinib group and 317 in the crizotinib group, with slight statistical heterogeneity between the studies (P = 0.49, I^2^ = 0%), but no obvious clinical heterogeneity, a fixed effect model was used for meta-analysis. Meta-analysis results shown in **Figure 9** suggest that the disease stability rate of the alectinib group was lower than that of the crizotinib group, and the difference was statistically significant [OR = 0.45, 95% CI (0.28, O.74), P = 0.001].

#### Adverse Events (AEs)

**Figure 10:**
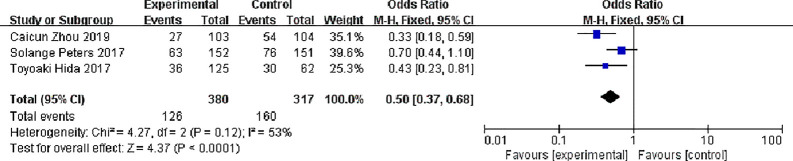
Meta-analysis of grades 3 to 5 adverse events between the alectinib group and crizotinib group.

Three studies have reported common adverse reactions in alectinib and Crizotinib, we focuses on the Grades 3 to 5 adverse events and we done a meta-analysis on grades 3 to 5 adverse events on alectinib and crizotinib, there were 380 patients in the alectinib group and 126 patients showed grades 3 to 5 adverse events while in Crizotinib group there were 317 patients and 160 patients showed grades 3 to 5 adverse events, with slight statistical heterogeneity between the studies (P = 0.12, I^2^ = 53%), but no obvious clinical heterogeneity the fixed effect model was used. Meta-analysis results shown in **Figure 10** suggest that the adverse events of alectinib group were lower than that of Crizotinib group, and the difference was statistically significant [OR = 0.50, 95% CI (0.23, 0.81), P = <0.0001].

## Discussion

Normally ALK inhibitors binds to the proteins pair of *EML4*-*ALK* fusion gene, ultimately break the signaling pathways (MAPK/STAT3/P13K/AKT) binds to fusion protein, resulting in decreased cellular proliferation. ALK inhibitors emerged as a key targeted gene therapy for the *ALK*-positive advance NSCLC in recent years. Crizotinib was the first ALK inhibitor approved for the treatment of *ALK*-positive NSCLC. In the foremost study on the clinical activity and safety of crizotinib soon after its development. In phase 1 study (35), 149 patients were enrolled, for the response-evaluable population 143 of whom were included; OR were seen in 87 (60.8%), including three complete responses and 84 partial responses. First documented objective response median time was 7.9 weeks (range 2.1–39.6) and median duration response was 49.1 weeks (95% CI 39.3–75.4); median PFS was 9.7 months (95% CI 7.7–12.8); estimated overall survival at 6 and 12 months was 87.9% (95% CI 81.3–92.3) and 74.8% (66.4–81.5). Overall 144 (97%) faced treatment-related adverse events, more of them were grade 1 or 2, most common AEs were visual effects, nausea, diarrhea, constipation, vomiting, and peripheral edema. Grade 3 or 4 AEs were neutropenia (n = 9), lymphopenia (n = 6), hypophosphatemia (n = 6), raised alanine aminotransferase (n = 6), results interpreted that Crizotinib is well tolerated with durable, rapid responses in patients with *ALK*-positive NSCLC.

PROFILE 1007, was conducted to evaluate crizotinib efficacy and safety in comparison with chemotherapy, 347 patients were included who had gotten one earlier platinum-based regimen. The primary end point was progression-free survival (PFS), results demonstrated that, median PFS in crizotinib was 7.7 *vs* 3.0 months in chemotherapy, HR 0.49; P <0.001, The response rate with crizotinib were 65% vs 20% with chemotherapy. Visual disorder, gastrointestinal side effects, and elevated liver aminotransferase levels were common adverse events related with crizotinib, while fatigue, alopecia, and dyspnea were common AEs with chemotherapy (36). The updated (PROFILE 1014) study which included 343 patients who had no any prior systemic treatment, primary end point was progression-free survival, PFS with crizotinib was 10.9 vs 7.0 months; HR 0.45; P <0.001; ORR 74% *vs* 45%; P <0.001. One-year survival probability was 84% *vs* 79%. Most common AEs, diarrhea, vision disorders, nausea, and edema were seen in patients receiving crizotinib, while adverse events associated with chemotherapy were nausea, vomiting, fatigue and decreased appetite (17). Though, patients getting crizotinib, regularly experience disease progression often within 12 months of starting treatment, partly due to secondary resistance mutations happening, additionally, due to poor blood–brain-barrier penetration of crizotinib (19, 20). Progression to the CNS is a common problem in patients with *ALK*-positive NSCLC treated with crizotinib. Thus, alternate ALK inhibitors, which have a dual function of high CNS efficacy and a wider range of overall survival against secondary *ALK* mutations were required.

Next generation, Alectinib is a highly selective oral ALK inhibitor, to evaluate its activity and safety, AF-001JP study, recruited ALK inhibitors naive patients, stage IIIB/IV, phase I part of the study approved dose of 300 mg twice per day in Japan. 46 patients in phase II part of the study received recommended dose, 43 achieved objective response (OR) 93.5% (95% CI, 82 to 99) of whom; two CRs (4·3%) and 41 PRs (89.1%). Grade 3 AEs were recorded as 26% and serious AEs were 11% (37). To further assess alectinib safety profile over a long administration period, 3 year follow-up (AF-001JP) study demonstrated that 18 of 46 patients had disease progression (39%); 3-year PFS 62%; (95% CI, 45 to 75); 3-year OS rate was 78% (13 events). At baseline 14 patients had brain metastasis, six patients in this study remained without CNS and systemic progression. Common treatment-related AE (all grades) was increased blood bilirubin (36.2%) (38). To assess its efficacy and safety in patients who have failed to prior crizotinib treatment, global phase 2 study (NP28673) included 138 patients from 16 countries, median age was 52 years, 80% had earlier chemotherapy, and at baseline 60% had CNS metastasis, median follow-up was 30 weeks, IRC assessed response in 122 with measurable disease at baseline exhibited OR of 49.2% (95% CI 40.0–58.4; all PRs); DCR 79.5%. In 96 patients with prior crizotinib or chemo; OR was 43.8%; DCR 78.1%. For 34 patients with measurable CNS disease at baseline, OR was 55.9% (including five CRs). Grades 3–5 AEs were observed in 27.5% (commonly, pulmonary embolism and dyspnea), dose interruptions 19.6%, reductions 8.7%, and withdrawals 8.0% (39). Another part of phase II (NP28761) study in US./Canadian population included 87 patients, median age was 54 y; 74% of the patients had prior chemotherapy; baseline CNS metastasis 55%; median follow-up 21 weeks. IRC-assessed response (69 with measurable disease at baseline) exhibited OR of 47.8% (95% CI 35.6–60.2); DCR 79.7%. In 16 patients with baseline CNS disease; OR was 68.8% (95% CI 41.3–89.0) including two CRs; DCR 100%. In 48 patients (with or without baseline CNS disease); DCR was 87.5% including nine CRs. Grades 3–5 AEs were observed in 31% (commonly; increased blood CPK, increased ALT, and increased AST). One patient had grade 5 hemorrhage. Dose interruptions were seen in (29%), reductions (14%), and withdrawals (2%) (40). Pooled overall survival and safety data from the pivotal phase II studies (NP28673 and NP28761). Pooled data of 225 patients exhibited that, 53.3% patients died at the final data cut-off time, 39.1% were alive and 7.6% withdrawn. Alectinib exhibit median overall survival (OS) 29.1 months (95% CI: 21.3–39.0) in the pooled analysis (NP28673 29.2 months [95% CI: 21.5–44.4]; NP28761 27.9 months [95% CI: 17.2–NE]). Mean dose intensity was 94.2%. Grade ≥3 adverse events occurred in 44.0%, common AEs included constipation (39.1%), fatigue (35.1%), peripheral edema (28.4%), myalgia (26.2%) and nausea (24.0%). Despite the longer treatment duration (median 48.6 weeks) alectinib demonstrated a tolerable safety profile consistent with previous studies. Dose reductions were seen in 14.7%, dose interruptions 37.3%, withdrawal 6.2% (41).

Depending upon the long term survival and safety in phase II trial and to evaluate its efficacy and safety in comparison with crizotinib on previously untreated patients, the first head-to-head comparative study on alectinib and crizotinib was conducted. In J-ALEX (phase 3) trial in Japanese patients, 207 patients were enrolled, alectinib (n = 103) and crizotinib (n = 104). Grade 3 or 4 AEs were lower with alectinib 26% *vs* 52% with crizotinib; Dose interruptions were also lower with alectinib 29% *vs* 74%; withdrawal with alectinib 9% *vs* 20% with crizotinib (12). In final PFS results of J-ALEX, median follow-up with alectinib was 42.4 *vs* 42.2 months with crizotinib, IRF-assessed PFS with alectinib shows HR 0.37, 95% CI: 0.26–0.52; median PFS 34.1 *vs* 10.2 months with crizotinib. Second interim OS analysis could not be concluded HR 0.80; P = 0.3860; median OS not estimable with alectinib *vs* 43.7 months crizotinib. Grade ≥3 AEs with alectinib were lower than crizotinib (36.9% *vs* 60.6%). OS follow-up continues (25). Another combined study for J-ALEX trial in western people included 303 patients (alectinib 152 *vs* 151 in crizotinib). During a median follow-up of (18.6 alectinib *vs* 17.6 months crizotinib) an event of disease progression or death ratio with alectinib were 41% versus 68% crizotinib. Investigator-assessed PFS (12 months events free) with alectinib 68.4% (95% CI 61.0 to 75.9) versus 48.7% (95% CI 40.4 to 56.9) in crizotinib; HR 0.47; P <0.001; median PFS not reached. Events of CNS progression with alectinib 12% *vs* 45% with crizotinib; HR 0.16; P <0.001. Overall response rate (ORR) with alectinib 82.9% (95% CI 76.0 to 88.5) *vs* 75.5 (95% CI 67.8 to 82.1) with crizotinib. Grades 3 to 5 AEs with alectinib were 41% *vs* 50% with crizotinib (13). In ALESIA (phase 3) study in Asian patients 187 patients were randomly enrolled (alectinib 125 *vs* 62 in crizotinib). Median follow-up with alectinib was 16.2 and 15.0 months in crizotinib. Investigator-assessed PFS in alectinib was significantly prolonged HR 0.22; P <0.0001; median PFS not estimable *vs* 11.1. IRC-PFS with alectinib was also prolonged HR 0.37; P <0.0001. OR with alectinib was 91% *vs* 48% with crizotinib, with a longer duration of response with alectinib versus crizotinib HR 0.22; P <0.0001. Objective response (OR) in baseline measurable or non-measurable CNS lesions were improved with alectinib 73% *vs* 22% with crizotinib. Grades 3 to 5 AEs with alectinib were lower despite of longer duration than crizotinib (29% *vs* 48%); serious AEs with alectinib were 15% *vs* 26% with crizotinib (11).

All the comparative studies showed significant results in favor of alectinib in terms of its efficacy and safety. Some experimental researches may also support the conclusion. Alectinib, as a highly selective ALK inhibitor, was specifically designed to overcome crizotinib resistance. In pre-clinical models, Alectinib overcome several gate-keeper mutations that impart resistance to Crizotinib like *ALK* L1196M mutation (42). The G1202R substitution that confers resistance to Alectinib is found in only approximately 2% of crizotinib-resistant patients (22). Unlike crizotinib, as evidence shown in *in vitro* studies, Alectinib is not a substrate of P-Glycoprotein (P-gp), which can promote the efflux of the blood–brain barrier (43). This could explain its higher ratio in cerebrospinal fluid (CSF) and significantly prolonged CNS PFS in clinical trials. This meta-analysis included three studies which are published in top international journals. The meta-analysis results showed that, Alectinib’s ORR, PFS, and PR are superior to crizotinib, and the side effects of Grades 3–5 are lower than crizotinib. Meta-analysis results provided an important basis for alectinib as the first-line drug for *ALK*-positive advanced NSCLC. For further enhancement in terms of overall survival and long term benefits whether the combination of alectinib and chemotherapy or the combination of alectinib and PD-L1 inhibitor can further improve the survival of patients.

In this meta-analysis only three studies are included but to confirm and analyze the sources of heterogeneity more clinical studies are needed. The advantages of this study are; first, the quality of the included literature is high and the results are highly reliable, secondly this is the first meta-analysis on the clinical efficacy and safety of alectinib versus crizotinib, which included the articles of high quality and are published in top ranked international journals. In this study, three articles are included (11–13), all international RCT studies; one article was published in the *Lancet Oncology* (12), one in *The New England Journal of Medicine* (13) and one in *Lancet Respiratory Medicine Journal* (11). In addition, this meta-analysis has certain limitations; ① There are few randomized controlled trials, comparing the efficacy and safety of alectinib versus crizotinib. ② Due to the high quality literature required for meta-analysis, there is lack of high quality published literature. To further elucidate the efficacy and safety of alectinib versus crizotinib, more randomized control trials on comparative studies of these two drugs are required.

## Conclusion

(1) For *ALK*-positive advanced NSCLC, alectinib is more effective than crizotinib.
(2) Compared with crizotinib, alectinib has a lower incidence of total adverse reactions.

## Data Availability Statement

The original contributions presented in the study are included in the article/**Supplementary Material**. Further inquiries can be directed to the corresponding author.

## Author Contributions

Study designing: ZM, HT, and LJ. Data search: ZM and HT. Data extraction: ZM and LJ. Data analysis and interpretation: ZM and ZZ. Manuscript drafting: HT and ZM. Manuscript critical intellectual content revision: ZM and ZJ. All authors contributed to the article and approved the submitted version.

## Funding

This work was supported by Hunan Province Science and Health Union Foundation (Nos. 2018JJ6135, 2018JJ6053).

## Conflict of Interest Statement

The authors declare that the research was conducted in the absence of any commercial or financial relationships that could be construed as a potential conflict of interest.
