# Supplementary material for: Comparison of Clinical Efficacy of Alectinib Versus Crizotinib in ALK-Positive Non-Small Cell Lung Cancer: A Meta-Analysis
Source: Front Oncol. 2021 Jun 2;11:646526. doi: 10.3389/fonc.2021.646526 (PMC8206528; doi:10.3389/fonc.2021.646526)
Supplement: Supplementary file 3 [file DataSheet_3.pdf]

Embase
Session Results
....

No.	Query Results	Results	Date
#35.	#20 AND #25 AND #33 AND #34	360	22 Apr 2021
#34.	#11 OR #19	406,446	22 Apr 2021
#33.	#26 OR #27 OR #28 OR #29 OR #30 OR #31 OR #32	7,529	22 Apr 2021
#32.	'xalkori' AND [1-1-1900]/sd NOT [16-2-2020]/sd AND [<1966-2020]/py	457	22 Apr 2021
#31.	pf02341066 AND [1-1-1900]/sd NOT [16-2-2020]/sd AND [<1966-2020]/py	139	22 Apr 2021
#30.	'pf 2341066' AND [1-1-1900]/sd NOT [16-2-2020]/sd AND [<1966-2020]/py	172	22 Apr 2021
#29.	'pf 02341066' AND [1-1-1900]/sd NOT [16-2-2020]/sd AND [<1966-2020]/py	442	22 Apr 2021
#28.	'pf-2341066' AND [1-1-1900]/sd NOT [16-2-2020]/sd AND [<1966-2020]/py	172	22 Apr 2021
#27.	'pf-02341066' AND [1-1-1900]/sd NOT [16-2-2020]/sd AND [<1966-2020]/py	442	22 Apr 2021
#26.	'crizotinib'/exp AND [1-1-1900]/sd NOT [16-2-2020]/sd AND [<1966-2020]/py	7,508	22 Apr 2021
#25.	#21 OR #22 OR #23 OR #24	1,545	22 Apr 2021
#24.	'ro5424802' AND [1-1-1900]/sd NOT [16-2-2020]/sd AND [<1966-2020]/py	20	22 Apr 2021
#23.	'ch5424802' AND [1-1-1900]/sd NOT [16-2-2020]/sd AND [<1966-2020]/py	60	22 Apr 2021
#22.	'alecensa' AND [1-1-1900]/sd NOT [16-2-2020]/sd AND [<1966-2020]/py	73	22 Apr 2021
#21.	'alectinib'/exp AND [1-1-1900]/sd NOT [16-2-2020]/sd AND [<1966-2020]/py	1,535	22 Apr 2021
#20.	'alk-positive' AND [1-1-1900]/sd NOT [16-2-2020]/sd AND [<1966-2020]/py	2,965	22 Apr 2021
#19.	#12 OR #13 OR #14 OR #15 OR #16 OR #17 OR #18	155,519	22 Apr 2021
#18.	'nonsmall cell lung cancer' AND [1-1-1900]/sd NOT [16-2-2020]/sd AND [<1966-2020]/py	4,622	22 Apr 2021
#17.	'non-small cell lung cancer' AND [1-1-1900]/sd NOT [16-2-2020]/sd AND [<1966-2020]/py	124,577	22 Apr 2021
#16.	'non-small cell lung carcinoma' AND [1-1-1900]/sd NOT [16-2-2020]/sd AND [<1966-2020]/py	6,243	22 Apr 2021
#15.	'non small cell lung carcinoma' AND [1-1-1900]/sd NOT [16-2-2020]/sd AND [<1966-2020]/py	6,243	22 Apr 2021
#14.	'non-small-cell lung carcinoma' AND [1-1-1900]/sd NOT [16-2-2020]/sd AND [<1966-2020]/py	6,243	22 Apr 2021

#13. 'non-small-cell lung carcinomas' AND [1-1-1900]/sd NOT [16-2-2020]/sd AND [<1966-2020]/py 1,317 22 Apr 2021

#12. 'non small cell lung cancer'/exp AND [1-1-1900]/sd NOT [16-2-2020]/sd AND [<1966-2020]/py 147,065 22 Apr 2021

#11. #1 OR #2 OR #3 OR #4 OR #5 OR #6 OR #7 OR #8 OR #9 OR #10 406,083 22 Apr 2021

#10. 'cancer of lung':ab,ti AND [1-1-1900]/sd NOT [16-2-2020]/sd AND [<1966-2020]/py 122 22 Apr 2021

#9. 'cancer of the lung':ab,ti AND [1-1-1900]/sd NOT [16-2-2020]/sd AND [<1966-2020]/py 2,152 22 Apr 2021

#8. 'pulmonary cancers' AND [1-1-1900]/sd NOT [16-2-2020]/sd AND [<1966-2020]/py 220 22 Apr 2021

#7. 'pulmonary cancer' AND [1-1-1900]/sd NOT [16-2-2020]/sd AND [<1966-2020]/py 1,595 22 Apr 2021

#6. 'lung cancers' AND [1-1-1900]/sd NOT [16-2-2020]/sd AND [<1966-2020]/py 17,044 22 Apr 2021

#5. 'lung cancer' AND [1-1-1900]/sd NOT [16-2-2020]/sd AND [<1966-2020]/py 316,138 22 Apr 2021

#4. 'pulmonary neoplasm' AND [1-1-1900]/sd NOT [16-2-2020]/sd AND [<1966-2020]/py 460 22 Apr 2021

#3. 'lung neoplasm' AND [1-1-1900]/sd NOT [16-2-2020]/sd AND [<1966-2020]/py 1,062 22 Apr 2021

#2. 'pulmonary neoplasms' AND [1-1-1900]/sd NOT [16-2-2020]/sd AND [<1966-2020]/py 735 22 Apr 2021

#1. 'lung cancer'/exp AND [1-1-1900]/sd NOT [16-2-2020]/sd AND [<1966-2020]/py 361,551 22 Apr 2021

……………………………………………

Cochrane Library

Search Name:
Date Run: 22/04/2021 12:38:19
Comment:

ID Search Hits
#1 MeSH descriptor: [Lung Neoplasms] explode all trees 7871
#2 MeSH descriptor: [Carcinoma, Non-Small-Cell Lung] explode all trees 4417
#3 (Lung):ti,ab,kw (Word variations have been searched) with Cochrane Library publication date Between Jan 1900 and Mar 2020 67134
#4 (Pulmo*):ti,ab,kw (Word variations have been searched) with Cochrane Library publication date Between Jan 1000 and Mar 2020 49914
#5 (neoplas*):ti,ab,kw (Word variations have been searched) with Cochrane Library publication date Between Jan 1000 and Mar 2020 78917

#6 (cancer):ti,ab,kw (Word variations have been searched) with Cochrane Library publication date Between Jan 1000 and Mar 2020 150874

#7 (carcinoma*):ti,ab,kw (Word variations have been searched) with Cochrane Library publication date Between Jan 1900 and Mar 2020 38896

#8 #3 OR #4 with Cochrane Library publication date Between Jan 1000 and Mar 2020 93715

#9 #5 OR #6 OR #7 with Cochrane Library publication date Between Jan 1000 and Mar 2020 179987

#10 #8 AND #9 with Cochrane Library publication date Between Jan 1000 and Mar 2020 26353

#11 #10 OR #1 OR #2 with Cochrane Library publication date Between Jan 1000 and Mar 2020 26455

#12 (Alectinib):ti,ab,kw (Word variations have been searched) with Cochrane Library publication date Between Jan 1000 and Mar 2020 103

#13 (Crizotinib):ti,ab,kw (Word variations have been searched) with Cochrane Library publication date Between Jan 1000 and Mar 2020 300

#14 (ALK-positive):ti,ab,kw (Word variations have been searched) with Cochrane Library publication date Between Jan 1000 and Mar 2020 182

#15 #11 AND #14 with Cochrane Library publication date Between Jan 1000 and Mar 2020 152

#16 (Alecensa):ti,ab,kw with Cochrane Library publication date Between Jan 1000 and Mar 2020 (Word variations have been searched) 4

#17 (CH5424802):ti,ab,kw with Cochrane Library publication date Between Jan 1000 and Mar 2020 (Word variations have been searched) 4

#18 (RO5424802):ti,ab,kw with Cochrane Library publication date Between Jan 1000 and Mar 2020 (Word variations have been searched) 12

#19 (PF-02341066):ti,ab,kw with Cochrane Library publication date Between Jan 1000 and Mar 2020 (Word variations have been searched) 10

#20 (PF-2341066):ti,ab,kw with Cochrane Library publication date Between Jan 1000 and Mar 2020 (Word variations have been searched) 0

#21 (PF 2341066):ti,ab,kw with Cochrane Library publication date Between Jan 1000 and Mar 2020 (Word variations have been searched) 0

#22 (PF2341066):ti,ab,kw with Cochrane Library publication date Between Jan 1000 and Mar 2020 (Word variations have been searched) 0

#23 (PF 02341066):ti,ab,kw with Cochrane Library publication date Between Jan 1000 and Mar 2020 (Word variations have been searched) 10

#24 (PF02341066):ti,ab,kw with Cochrane Library publication date Between Jan 1000 and Mar 2020 (Word variations have been searched) 0

#25 (Xalkori):ti,ab,kw with Cochrane Library publication date Between Jan 1000 and Mar 2020 (Word variations have been searched) 8

#26 #12 OR #16 OR #17 OR #18 106

#27 #13 OR #19 OR #20 OR #21 OR #22 OR #23 OR #24 OR #25 300

#28 #15 AND #26 AND #27 46
