# Supplementary material for: Comparison of Clinical Efficacy of Alectinib Versus Crizotinib in ALK-Positive Non-Small Cell Lung Cancer: A Meta-Analysis
Source: Front Oncol. 2021 Jun 2;11:646526. doi: 10.3389/fonc.2021.646526 (PMC8206528; doi:10.3389/fonc.2021.646526)
Supplement: Supplementary file 4 [file DataSheet_4.zip › Supplementary materialsú║search strategy and results/Embase queries & Cochrane Library_search_manager_search.docx]

Embase

Session Results

.......................................................

No. Query Results Results Date

#35. #20 AND #25 AND #33 AND #34 360 22 Apr 2021

#34. #11 OR #19 406,446 22 Apr 2021

#33. #26 OR #27 OR #28 OR #29 OR #30 OR #31 OR #32 7,529 22 Apr 2021

#32. 'xalkori' AND [1-1-1900]/sd NOT [16-2-2020]/sd 457 22 Apr 2021

AND [<1966-2020]/py

#31. pf02341066 AND [1-1-1900]/sd NOT [16-2-2020]/sd 139 22 Apr 2021

AND [<1966-2020]/py

#30. 'pf 2341066' AND [1-1-1900]/sd NOT [16-2-2020]/sd 172 22 Apr 2021

AND [<1966-2020]/py

#29. 'pf 02341066' AND [1-1-1900]/sd NOT 442 22 Apr 2021

[16-2-2020]/sd AND [<1966-2020]/py

#28. 'pf-2341066' AND [1-1-1900]/sd NOT [16-2-2020]/sd 172 22 Apr 2021

AND [<1966-2020]/py

#27. 'pf-02341066' AND [1-1-1900]/sd NOT 442 22 Apr 2021

[16-2-2020]/sd AND [<1966-2020]/py

#26. 'crizotinib'/exp AND [1-1-1900]/sd NOT 7,508 22 Apr 2021

[16-2-2020]/sd AND [<1966-2020]/py

#25. #21 OR #22 OR #23 OR #24 1,545 22 Apr 2021

#24. 'ro5424802' AND [1-1-1900]/sd NOT [16-2-2020]/sd 20 22 Apr 2021

AND [<1966-2020]/py

#23. 'ch5424802' AND [1-1-1900]/sd NOT [16-2-2020]/sd 60 22 Apr 2021

AND [<1966-2020]/py

#22. 'alecensa' AND [1-1-1900]/sd NOT [16-2-2020]/sd 73 22 Apr 2021

AND [<1966-2020]/py

#21. 'alectinib'/exp AND [1-1-1900]/sd NOT 1,535 22 Apr 2021

[16-2-2020]/sd AND [<1966-2020]/py

#20. 'alk-positive' AND [1-1-1900]/sd NOT 2,965 22 Apr 2021

[16-2-2020]/sd AND [<1966-2020]/py

#19. #12 OR #13 OR #14 OR #15 OR #16 OR #17 OR #18 155,519 22 Apr 2021

#18. 'nonsmall cell lung cancer' AND [1-1-1900]/sd NOT 4,622 22 Apr 2021

[16-2-2020]/sd AND [<1966-2020]/py

#17. 'non-small cell lung cancer' AND [1-1-1900]/sd 124,577 22 Apr 2021

NOT [16-2-2020]/sd AND [<1966-2020]/py

#16. 'non-small cell lung carcinoma' AND [1-1-1900]/sd 6,243 22 Apr 2021

NOT [16-2-2020]/sd AND [<1966-2020]/py

#15. 'non small cell lung carcinoma' AND [1-1-1900]/sd 6,243 22 Apr 2021

NOT [16-2-2020]/sd AND [<1966-2020]/py

#14. 'non-small-cell lung carcinoma' AND [1-1-1900]/sd 6,243 22 Apr 2021

NOT [16-2-2020]/sd AND [<1966-2020]/py

#13. 'non-small-cell lung carcinomas' AND 1,317 22 Apr 2021

[1-1-1900]/sd NOT [16-2-2020]/sd AND

[<1966-2020]/py

#12. 'non small cell lung cancer'/exp AND 147,065 22 Apr 2021

[1-1-1900]/sd NOT [16-2-2020]/sd AND

[<1966-2020]/py

#11. #1 OR #2 OR #3 OR #4 OR #5 OR #6 OR #7 OR #8 OR 406,083 22 Apr 2021

#9 OR #10

#10. 'cancer of lung':ab,ti AND [1-1-1900]/sd NOT 122 22 Apr 2021

[16-2-2020]/sd AND [<1966-2020]/py

#9. 'cancer of the lung':ab,ti AND [1-1-1900]/sd NOT 2,152 22 Apr 2021

[16-2-2020]/sd AND [<1966-2020]/py

#8. 'pulmonary cancers' AND [1-1-1900]/sd NOT 220 22 Apr 2021

[16-2-2020]/sd AND [<1966-2020]/py

#7. 'pulmonary cancer' AND [1-1-1900]/sd NOT 1,595 22 Apr 2021

[16-2-2020]/sd AND [<1966-2020]/py

#6. 'lung cancers' AND [1-1-1900]/sd NOT 17,044 22 Apr 2021

[16-2-2020]/sd AND [<1966-2020]/py

#5. 'lung cancer' AND [1-1-1900]/sd NOT 316,138 22 Apr 2021

[16-2-2020]/sd AND [<1966-2020]/py

#4. 'pulmonary neoplasm' AND [1-1-1900]/sd NOT 460 22 Apr 2021

[16-2-2020]/sd AND [<1966-2020]/py

#3. 'lung neoplasm' AND [1-1-1900]/sd NOT 1,062 22 Apr 2021

[16-2-2020]/sd AND [<1966-2020]/py

#2. 'pulmonary neoplasms' AND [1-1-1900]/sd NOT 735 22 Apr 2021

[16-2-2020]/sd AND [<1966-2020]/py

#1. 'lung cancer'/exp AND [1-1-1900]/sd NOT 361,551 22 Apr 2021

[16-2-2020]/sd AND [<1966-2020]/py

.......................................................

Cochrane Library

Search Name:

Date Run: 22/04/2021 12:38:19

Comment:

ID Search Hits

#1 MeSH descriptor: [Lung Neoplasms] explode all trees 7871

#2 MeSH descriptor: [Carcinoma, Non-Small-Cell Lung] explode all trees 4417

#3 (Lung):ti,ab,kw (Word variations have been searched) with Cochrane Library publication date Between Jan 1900 and Mar 2020 67134

#4 (Pulmo*):ti,ab,kw (Word variations have been searched) with Cochrane Library publication date Between Jan 1000 and Mar 2020 49914

#5 (neoplas*):ti,ab,kw (Word variations have been searched) with Cochrane Library publication date Between Jan 1000 and Mar 2020 78917

#6 (cancer):ti,ab,kw (Word variations have been searched) with Cochrane Library publication date Between Jan 1000 and Mar 2020 150874

#7 (carcinoma*):ti,ab,kw (Word variations have been searched) with Cochrane Library publication date Between Jan 1900 and Mar 2020 38896

#8 #3 OR #4 with Cochrane Library publication date Between Jan 1000 and Mar 2020 93715

#9 #5 OR #6 OR #7 with Cochrane Library publication date Between Jan 1000 and Mar 2020 179987

#10 #8 AND #9 with Cochrane Library publication date Between Jan 1000 and Mar 2020 26353

#11 #10 OR #1 OR #2 with Cochrane Library publication date Between Jan 1000 and Mar 2020 26455

#12 (Alectinib):ti,ab,kw (Word variations have been searched) with Cochrane Library publication date Between Jan 1000 and Mar 2020 103

#13 (Crizotinib):ti,ab,kw (Word variations have been searched) with Cochrane Library publication date Between Jan 1000 and Mar 2020 300

#14 (ALK-positive):ti,ab,kw (Word variations have been searched) with Cochrane Library publication date Between Jan 1000 and Mar 2020 182

#15 #11 AND #14 with Cochrane Library publication date Between Jan 1000 and Mar 2020 152

#16 (Alecensa):ti,ab,kw with Cochrane Library publication date Between Jan 1000 and Mar 2020 (Word variations have been searched) 4

#17 (CH5424802):ti,ab,kw with Cochrane Library publication date Between Jan 1000 and Mar 2020 (Word variations have been searched) 4

#18 (RO5424802):ti,ab,kw with Cochrane Library publication date Between Jan 1000 and Mar 2020 (Word variations have been searched) 12

#19 (PF-02341066):ti,ab,kw with Cochrane Library publication date Between Jan 1000 and Mar 2020 (Word variations have been searched) 10

#20 (PF-2341066):ti,ab,kw with Cochrane Library publication date Between Jan 1000 and Mar 2020 (Word variations have been searched) 0

#21 (PF 2341066):ti,ab,kw with Cochrane Library publication date Between Jan 1000 and Mar 2020 (Word variations have been searched) 0

#22 (PF2341066):ti,ab,kw with Cochrane Library publication date Between Jan 1000 and Mar 2020 (Word variations have been searched) 0

#23 (PF 02341066):ti,ab,kw with Cochrane Library publication date Between Jan 1000 and Mar 2020 (Word variations have been searched) 10

#24 (PF02341066):ti,ab,kw with Cochrane Library publication date Between Jan 1000 and Mar 2020 (Word variations have been searched) 0

#25 (Xalkori):ti,ab,kw with Cochrane Library publication date Between Jan 1000 and Mar 2020 (Word variations have been searched) 8

#26 #12 OR #16 OR #17 OR #18 106

#27 #13 OR #19 OR #20 OR #21 OR #22 OR #23 OR #24 OR #25 300

#28 #15 AND #26 AND #27 46
